# Acute, Subchronic, and Chronic Complications of Radical Prostatectomy Versus Radiotherapy With Hormone Therapy in Older Adults With High-Risk Prostate Adenocarcinoma

**DOI:** 10.3389/fonc.2022.875036

**Published:** 2022-05-02

**Authors:** Szu-Yuan Wu, Le Duc Huy, Chih Jung Liao, Chung-Chien Huang

**Affiliations:** ^1^ Department of Food Nutrition and Health Biotechnology, College of Medical and Health Science, Asia University, Taichung, Taiwan; ^2^ Big Data Center, Lo-Hsu Medical Foundation, Lotung Poh-Ai Hospital, Yilan, Taiwan; ^3^ Division of Radiation Oncology, Lo-Hsu Medical Foundation, Lotung Poh-Ai Hospital, Yilan, Taiwan; ^4^ Department of Healthcare Administration, College of Medical and Health Science, Asia University, Taichung, Taiwan; ^5^ Cancer Center, Lo-Hsu Medical Foundation, Lotung Poh-Ai Hospital, Yilan, Taiwan; ^6^ Graduate Institute of Business Administration, College of Management, Fu Jen Catholic University, Taipei, Taiwan; ^7^ Centers for Regional Anesthesia and Pain Medicine, Taipei Municipal Wan Fang Hospital, Taipei Medical University, Taipei, Taiwan; ^8^ Master Program in School of Health Care Administration, Department of Health Care Administration, College of Management, Taipei Medical University, Taipei, Taiwan; ^9^ Health Management Training Institute, University of Medicine and Pharmacy, Hue University, Hue, Vietnam; ^10^ Department of Medical Imaging, Taipei Medical University-Shuang-ho Hospital, New Taipei City, Taiwan; ^11^ International PhD Program in Biotech and Healthcare Management, School of Health Care Administration, College of Management, Taipei Medical University, Taipei, Taiwan; ^12^ Department of Medical Quality, Taipei Municipal Wan Fang Hospital - Managed by Taipei Medical University, Taipei, Taiwan; ^13^ Department of Long-Term Care and School of Gerontology Health Management, College of Nursing, Taipei Medical University, Taipei, Taiwan; ^14^ Department and School of Pharmacy, College of Pharmacy, Taipei Medical University, Taipei, Taiwan

**Keywords:** complications, old age, high-risk localized prostate cancer, radical prostatectomy, intensity-modulated radiotherapy

## Abstract

**Purpose:**

To compare acute, subchronic, and chronic complications between older patients with high-risk localized prostate cancer (HR-LPC) receiving radical prostatectomy (RP) and high-dose intensity-modulated radiotherapy (IMRT) combined with long-term hormone therapy (HT).

**Patients and Methods:**

We recruited older patients (≥80 years) with HR-LPC from the Taiwan Cancer Registry database. After propensity score matching, logistic regression analysis was used to compare the acute, subchronic, and chronic complication rates between patients who underwent RP (the RP group) and high-dose IMRT combined with long-term HT (the IMRT+HT group).

**Results:**

Benign prostatic hyperplasia (BPH) symptoms and urinary incontinence (UI) were the most common complications over 5 years (BPH symptoms: RP, 17.69%; IMRT+HT, 29.58%; UI: RP, 10.47%; IMRT+HT, 5.50%). Compared with the RP group, the IMRT+HT group had higher odds of BPH symptoms and lower odds of UI and hernia after the 5-year follow-up period. The impotence rates were significantly higher in the IMRT+HT group than in the RP group at 3 months and 1 year after treatment and became nonsignificant after 2 years. At 5 years after treatment, the IMRT+HT group had lower risks of UI (adjusted odds ratio [aOR], 0.50; 95% confidence interval [CI], 0.28–0.88) and hernia (aOR, 0.21; 95% CI, 0.11–0.82) and a higher risk of BPH symptoms (aOR, 4.15; 95% CI, 2.82–7.37) than the RP group.

**Conclusion:**

IMRT+HT was associated with lower UI and hernia risks than RP. By contrast, RP was associated with fewer complications of BPH over the follow-up period and less impotence during the first year after treatment. Our findings provide important and valuable references for shared decision-making for optimal therapy selection among older men with HR-LPC.

## Introduction

According to the Global Cancer Observatory data in 2020, prostate cancer (PC) was the leading cause of cancer and the second leading cause of death among men aged ≥70 years.(Organization) One in five older men in Western countries have PC, and PC-related mortality rate among older men (aged >75 years) has reached nearly 70%, especially in the high-risk group of PC, with 45% PC death at 10 years (1, 2). In Taiwan, PC is the sixth leading cause of cancer death (3) and accounts for over one-fifth of newly diagnosed PC cases per year in men older than 80 years (4). Understanding the treatments of high-risk localized prostate cancer (HR-LPC)–related complications is increasingly important for shared clinical decision-making by older patients and physicians because active surveillance for older men with HR-LPC and life expectancy >5 years might be high risk for cancer mortality (1, 2).

Accumulating evidence demonstrates the increasing incidence of HR-LPC in older men (1, 2, 5). In the United States, older men with PC are more likely to have a high-risk disease and low overall and cancer-specific survival rates (6). Between 2014 and 2015, large-scale epidemiological surveillance conducted by the US Preventive Services Task Force indicated a declining trend in the incidence of low-risk disease and a considerably increasing trend of intermediate-, high-, and very high-risk PC among men older than 75 years (7). However, few studies have evaluated therapeutic interventions in this age group; older men are often excluded from clinical trials (2) because of concerns regarding significant differences between older and younger patients in drug pharmacokinetics and pharmacodynamics, comorbidities, and treatment-related complications (8–10).

To decide the primary therapy in patients, physicians need to stratify the risk of PC and consider life expectancy (11). In Taiwan, most oncological physicians conduct risk assessment based on National Comprehensive Cancer Network (NCCN) guidelines (11–15). NCCN guidelines recommend that risk stratification should be based on metastatic progression, pretreatment levels of serum prostate-specific antigen (PSA), initial Gleason score, grade based on the initial biopsy, and extent of cancer involvement (clinical tumor [T] stages) (11). In patients with HR-LPC (diagnosed as per NCCN guidelines) and life expectancy over 5 years, the recommended treatment is either radical prostatectomy (RP) or a combination of high-dose (≥72 Gy) external beam radiotherapy (EBRT) (16) plus long-term (≥1.5 years) androgen-deprivation therapy (ADT) (11, 17). However, which therapy is optimum for older patients, particularly those with HR-LPC, remains unclear. In addition, strong evidence is lacking because most clinical trials have a small sample size; have inadequately adjusted for various parameters related to health, age, or lymph node evaluation; and lack consistent risk stratification and treatments such as radiation dose, EBRT techniques, or duration of ADT use (18–23). Moreover, although several studies have investigated the adverse effects of RP and EBRT on younger men (23, 24), the long-term outcomes in older patients, especially those with HR-LPC, undergoing RP or intensity-modulated radiotherapy (IMRT, one advanced technique of EBRT) combined with long-term hormone therapy (HT) are unclear. Therefore, we compared acute, subchronic, and chronic complications in older men with HR-LPC undergoing RP or high-dose IMRT combined with long-term HT.

## Patients and Methods

### Database

This population-based retrospective cohort study compared acute, subchronic, or chronic postoperative complications in older men with HR-LPC undergoing open RP or high-dose IMRT combined with long-term HT. All data were retrieved from the nationwide Taiwan Cancer Registry Database (TCRD), which contains the data of over 97% of patients with cancer in Taiwan since 1979 (12–15, 24–26). The detailed information, including clinical stages, surgical techniques, radiotherapy, hormone treatments, pathologic stages, and follow-up visits, was accessed from the TCRD of the Collaboration Center of Health Information Application (12, 14, 15, 26). On the basis of encrypted patient identifiers, the data in TCRD were linked with Taiwan’s National Health Insurance Research Database (NHIRD). The NHIRD contains all medical claims data on disease diagnoses, demographic characteristics, procedures, medication records, and enrollment profiles of all beneficiaries (25). The study protocols were reviewed and approved by the Institutional Review Board of Tzu-Chi Medical Foundation (IRB109-015-B).

### Study Cohort

By using the merged data of NHIRD and TCRD, we included men ≥80 years old with HR-LPC who underwent either open RP (the RP group) or high-dose IMRT combined with the long-term (18–36 months) HT (the IMRT+HT group) between January 1, 2011, and December 31, 2016. The index date was the confirmation date of PC diagnosis based on pathological results. Patients were followed up until December 31, 2018.

The diagnosis of HR-LPC was verified through a review of pathological data, magnetic resonance images of PC staging (T1–T3a), preoperative PSA levels (0–20 ng/mL), or grade (1–5) based on NCCN risk groups (11). In addition, the data of patients with newly diagnosed PC, who had no prior RP or IMRT+HT, were reviewed to ensure no other neoplasm, clinical lymph node metastasis, or distant metastasis. We excluded participants with missing data on clinical or pathological stage, D’Amico risk classification, Gleason score, postoperative Gleason grade, or preoperative PSA concentration, as well as those with clinical node-positive PC, nonadenocarcinoma histology, and life expectancy < 5 years.

RP was defined as a surgical procedure to resect the entire prostate gland and the surrounding lymph nodes for men with localized PC (27). For high-dose IMRT, the prophylactic doses of 1.8–45 Gy per fraction and 54 Gy were used for the pelvic lymph node and seminal vesicles, respectively, whereas cone-down boosts of 72–81 Gy (median: 75.6 Gy) were employed to cover the prostate (16, 19). To control for potential bias, we excluded patients with any of the following features: receiving an insufficient dose of IMRT (<72 Gy) (16), undergoing IMRT alone or with short-term HT (<18 months) (11), and receiving lower standard RP.

### Study Covariates and End Points

Our independent variables were therapy modalities (RP or IMRT+HT), age, diagnosis year, income, hospital area, hospital level (academic or nonacademic), clinical T stage, grade (maximum Gleason grade), pretreatment PSA (ng/mL), and D’Amico risk classification. The endpoints were acute (in the third month), subchronic complications (at 1 and 2 years), and late complications (at 3, 4, and 5 years) after receiving treatments (i.e., symptoms of benign prostatic hyperplasia [BPH], impotence, urinary incontinence [UI], urethral stricture, and hernia) (13, 28). Symptoms of BPH, impotence, UI, urethral stricture, and hernia were recorded in medical records and treatments were accordance with the corresponding medical treatments. The records of BPH, impotence, UI, urethral stricture, and hernia were similar with the previous study (13). Patients with impotence, UI, urethral stricture, and hernia before the index date were excluded from this study. We did not exclude men with BPH before the index date because older patients with LPC have a very high prevalence (approximately 70%) of BPH (29).

### PSM

We conducted head-to-head PSM at a 1:2 ratio between the RP and high-dose IMRT plus long-term HT groups (30). However, only some independent variables were matched at this ratio, whereas other variables were matched at 1:2 or 1:1.

### Statistical Analysis

Normally distributed continuous variables are presented as mean (standard deviation [SD]), nonnormally distributed continuous variables as median (interquartile range), and categorical variables as number (percentage). Depending on data characteristics, appropriate comparison tests (*t* test, analysis of variance, and nonparametric tests) were selected.

After PSM, logistic regression models were fitted to examine the association between therapeutic modalities (RP and IMRT+HT) and posttreatment outcomes, including BPH symptoms, impotence, UI, urethral stricture, hernia. The models were also adjusted for other covariates: age, year of diagnosis, CCI score, myocardial infarction, congestive heart failure, Peripheral vascular disease, comorbidities (cerebrovascular disease, chronic obstructive pulmonary disease, diabetes, and hypertension), income, hospital area, hospital level, clinical T stage, Gleason score, grade, pretreatment PSA, and D’Amico risk classification. A two-tailed *P* value of <.05 was set as statistically significant. All analyses were conducted using SAS version 9.4 (SAS Institute, Cary, NC, USA).

## Results

### Study Cohort

The sample size were 659 patients after PSM with unmatched excluded who were eligible for further analysis. Among 659 older patients with HR-LPC, 277 (42.0%) underwent RP (mean [SD] age, 84.6 [3.2] years), and 382 (58.0%) received high-dose IMRT+HT (mean [SD] age, 85.1 [3.5] years) (**Table S1**). The mean [SD] follow-up period of the RP and IMRT+HT groups from the index date was 60.2 [17.6] months and 59.9 [17.3] months, respectively. No significant differences were observed in confounders (age, year of diagnosis, CCI score, chronic conditions, income, hospital level, clinical T stage, Gleason score, grade, pretreatment PSA concentration, D’Amico risk stratification, and follow-up duration) (**Table S1**).

Overall, BPH symptoms and UI were the most common chronic complications over 5 years after treatment in both groups (BPH symptoms: RP, 46 [17.69%]; IMRT+HT, 113 [29.58%]; UI: RP, 29 [10.47%]; IMRT+HT, 21 [5.50%]) (**Table 2**). Compared with the RP group, the IMRT+HT group had higher odds of BPH symptoms and lower odds of UI and hernia over the 5-year follow-up period. The acute, subchronic, and chronic impotence rates were significantly different between the two groups at 3 months and 1 year after treatment and became nonsignificant after 2 years (**Tables 1**, **2**).

**Table 1:** Logistic regression analysis of the association of therapeutic modalities with acute and subchronic complications for older men aged ≥80 years with high-risk prostate adenocarcinoma.

	RP	IMRT + long-term HT		IMRT vs RP (ref.)		
	N = 277, %	N = 382, %		adjusted odds ratios^*^ (95% CI)		*P* value
**Days 1–90 after Tx**						
BPH symptoms	264 (88.81)	376 (98.43)		3.09 (1.21, 7.87)	.022
Impotence	21 (7.58)	31 (8.12)		1.17 (1.03, 1.35)	.035
UI	99 (35.74)	76 (19.90)		0.45 (0.32, 0.63)	.007
Urethral strictures	12 (4.33)	13 (3.40)		1.18 (0.34, 1.76)	.391
Hernias	30 (10.83)	16 (4.19)		0.36 (0.19, 0.68)	.017
**Days 91–365 after Tx**						
BPH symptoms	128 (46.21)	332 (86.91)		6.05 (4.53, 9.72)	.009
Impotence	9 (3.25)	26 (6.81)		1.19 (1.01, 2.81)	.045
UI	70 (25.27)	72 (18.85)		0.69 (0.48, 0.97)	.048
Urethral strictures	6 (2.17)	6 (1.57)		1.21 (0.80, 7.25)	.262
Hernias	28 (10.11)	13 (3.40)		0.32 (0.16, 0.62)	.021
**At 2 years after Tx**						
BPH symptoms	113 (40.79)	310 (81.15)		6.35 (5.02, 9.76)	.003
Impotence	11 (3.97)	20 (5.24)		1.50 (0.70, 3.19)	.359
UI	72 (25.99)	68 (17.80)		0.62 (0.43, 0.92)	.034
Urethral strictures	8 (2.89)	7 (1.83)		1.65 (0.38, 8.29)	.184
Hernias	30 (10.83)	13 (3.40)		0.29 (0.15, 0.58)	.005

^*^Multivariable model with adjustment of covariates: Age, year of diagnosis, CCI scores, myocardial infarction, congestive heart failure, peripheral vascular disease, cerebrovascular disease, chronic pulmonary disease, diabetes, hypertension, income, hospital area, hospital level, clinical T stages, Gleason score, grade, preoperative PSA, and D’Amico risk classification. Least square mean difference for odds ratio for binary variables via fitting generalized liner model with stratification of matched pairs.

RP, radical prostatectomy; IMRT, intensity-modulated radiation therapy; CI, confidence interval; UI, urinary incontinence; Ref., reference group; N, number; BPH, benign prostatic hyperplasia; HT, hormone therapy; Tx, treatment.

**Table 2:** Logistic regression analysis of the association of therapeutic modalities with late complications for older men aged ≥80 years with high-risk prostate adenocarcinoma.

	RP	IMRT+ long-term HT	IMRT vs RP (ref.)		
	N = 277, %	N = 382, %	adjusted odds ratios^*^ (95% CI)		*P* value
**At 3 years after Tx**					
BPH symptoms	105 (37.91)	276 (72.25)	5.68 (3.91, 8.25)	.021	
Impotence	11 (3.97)	15 (3.93)	1.47 (0.71, 3.08)	.252	
UI	60 (21.66)	50 (13.09)	0.69 (0.46, 0.84)	.007	
Urethral strictures	5 (1.81)	5 (1.31)	1.97 (0.87, 8.57)	.237	
Hernias	20 (7.22)	9 (2.36)	0.32 (0.15, 0.71)	.014	
**At 4 years after Tx**					
BPH symptoms	58 (20.94)	142 (37.17)	5.19 (3.52, 9.21)	.009	
Impotence	6 (2.17)	10 (2.61)	1.26 (0.73, 5.47)	.121	
UI	36 (13.00)	26 (6.81)	0.51 (0.29, 0.89)	.013	
Urethral strictures	3 (1.08)	3 (0.79)	1.04 (0.57, 2.23)	.321	
Hernias	10 (3.61)	3 (0.79)	0.23 (0.16, 0.85)	.005	
**At 5 years after Tx**					
BPH symptoms	46 (17.69)	113 (29.58)	4.15 (2.82, 7.37)	.007	
Impotence	5 (1.81)	8 (2.09)	1.02 (0.72, 2.11)	.118	
UI	29 (10.47)	21 (5.50)	0.50 (0.28, 0.88)	.008	
Urethral strictures	2 (0.72)	2 (0.52)	1.01 (0.55, 2.01)	.230	
Hernias	8 (2.89)	2 (0.52)	0.21 (0.11, 0.82)	.002	

^*^Multivariable model with adjustment of covariates: Age, year of diagnosis, CCI scores, myocardial infarction, congestive heart failure, peripheral vascular disease, cerebrovascular disease, chronic pulmonary disease, diabetes, hypertension, income, hospital area, hospital level, clinical T stages, Gleason score, grade, preoperative PSA, and D’Amico risk classification. Least square mean difference for odds ratio for binary variables via fitting generalized linear model with stratification of matched pairs.

RP, radical prostatectomy; IMRT, intensity-modulated radiation therapy; CI, confidence interval; UI, urinary incontinence; Ref., reference group; N, number; BPH, benign prostatic hyperplasia; Tx, treatment.

### Acute and Subchronic Complications

For both therapeutic modalities, the BPH symptoms were the most common subchronic complication during the first 2 years after treatment (RP, 113 [40.79%]; IMRT+HT, 310 [81.15%]) (**Table 1**). In the RP group, the incidence of acute and subchronic complications decreased considerably in the first year and then slightly increased in the second posttreatment year; the incidence in the IMRT+HT group decreased continuously over 2 years of therapy. In the adjusted logistic regression model, the odds ratio of UI and hernia were lower in patients receiving IMRT+HT than in patients undergoing RP over 2 years after treatment (**Table 1**). In year 2, compared with the RP group, the aORs of the IMRT+HT group were 0.62 (95% confidence interval [CI], 0.43–0.92) for UI and 0.29 (95% CI, 0.15–0.58) for hernia. Regarding impotence, the odds ratios of patients who received IMRT+HT therapy were significant in the third month (aOR, 1.17; 95% CI, 1.03–1.35) and the first year (aOR, 1.19; 95% CI, 1.01–2.81) after treatment and became nonsignificant in the second postoperative year (aOR, 1.50; 95% CI, 0.70–3.19).

### Chronic Complications After Treatment

Overall, fewer patients had late complications in both therapy types between 3 and 5 years, such as BPH symptoms, impotence, UI, urethral stricture, and hernia (**Table 2**). Similar to the previous period, the odds of UI and hernia were lower in the IMRT+HT group than in the RP group at 3–5 years after treatment, whereas the IMRT+HT group had a higher risk of BPH symptoms than the RP group (**Table 2**). A 4 years after treatment, the incidence of BPH in the IMRT+HT group decreased considerably by more than 48% compared with that in the previous year (adjusted odds ratio [aOR], 5.19; 95% CI, 3.52–9.21). At 5 years after treatment, the IMRT+HT group had lower risks of UI (aOR, 0.50; 95% CI, 0.28–0.88) and hernia (aOR, 0.21; 95% CI, 0.11–0.82) and a higher risk of BPH symptoms (aOR, 4.15; 95% CI, 2.82–7.37) than the RP group.

## Discussion

According to NCCN guidelines, either RP or EBRT combined with long-term HT is effective for treating patients diagnosed with HR-LPC (11). The decision of optimal therapy relies on different factors, such as clinical stage, pathologic grade, PSA level, and comorbidity-adjusted life expectancy (11, 31). Although several studies have indicated the equivalent effects of RP or RT on oncological outcomes in patients with LPC (15, 32), comparative studies on acute and chronic adverse effects of RP and RT in different age groups with risk stratification are scant. One of the most prominent studies comparing long-term outcomes between ERBT combined with HT and RP compared patient-reported outcomes after treatment in patients from the ProtecT trial (23). However, the researchers did not include risk stratification, old age, RT technique, and long-term ADT in their analysis (9). A recent study by Hoffman partly addressed this gap by comparing long-term posttreatment outcomes of these treatments between unfavorable and favorable condition groups; however, their study population was limited to patients 59–70 years old (33). To the best of our knowledge, ours is the first study to compare posttreatments outcomes in men older than 80 years with HR-LPC undergoing either RP or IMRT+HT.

Most previous randomized control trials (RCTs) and population-based cohort studies have recruited only a small sample of Asian patients and older men with HR-LPC, precluding the generalizability of their results (32, 34, 35). In the ProtecT trial, White people accounted for nearly 99% of the study population (23). The target population in the PCOS study was Hispanic or non-Hispanic individuals, and Asians were not included in the study (34). The findings of our study comparing acute, subchronic, and chronic complications of different HR-LPC treatments (RP versus high-dose IMRT+long-term HT) should guide shared decision-making for older men with HR-LPC, especially in those in the Asian population.

Patients with LPC who receive RP are more likely to experience UI and erectile dysfunction than those undergoing RT with or without HT (23, 24, 33, 34). However, no study has compared acute, subchronic, and chronic posttreatment complications, such as BPH symptoms, UI, impotence, urethral stricture, and hernia, according to RP and IMRT+HT in men older than 80 years with HR-LPC. Ours is the first study to compare the incidence of acute, subchronic, and chronic posttreatment complications in older men with HR-LPC.

Our analysis indicated that over the 5-year follow-up period, except for BPH symptoms and impotence, the RP group had a higher complication rate in terms of UI and hernia than the IMRT+HT group (**Tables 1**, **2**). Moreover, the prevalence of impotence in the IMRT+HT group was higher than that in the RP group. This finding is inconsistent with those of previous studies (23, 33–35). Hoffman et al. concluded that unfavorable-risk patients with a young age undergoing prostatectomy exhibited worse 5-year sexual function outcomes than those undergoing EBRT (33). The ProtecT trial with unclear risk groups provided a similar finding that surgical therapy negatively influenced sexual function, particularly erectile function during the first 6 months, and the effect was worse than that in the other treatment groups over 6 years (23). The higher 2-year impotence rate in the IMRT+HT group in our cohort may be attributed to long-term (1.5 years at least) HT use in men with HR-LPC. Moreover, the difference between the RP and IMRT+HT groups became nonsignificant after 2 years, which is compatible with the result of the ProtecT trial (**Tables 1**, **2**) (23). Another possible explanation is that the previous studies focused on a younger population and did not evaluate disease severity; additionally, most men did not have HR-LPC in the previous studies (23, 33). Zattoni et al. emphasized that sexual function should not be a posttreatment outcome in older patients because they are more likely to experience decreasing sexual function over time, with a low chance of posttreatment recovery (36). Sexual dysfunction is a common problem among older patients after 75 years old (37, 38).

Our study also indicated that patients in the RP group were more likely to experience UI than those in the IMRT+HT group during the 5-year follow-up (**Table 2**). This finding is compatible with those of previous studies (23, 24, 34) and may be because patients with HR-LPC receive more extensive surgery. Untill now, UI is one of the most few sequelae of RP (39, 40). Therefore, different surgical modifications, to restore the original anatomy, were proposed to overcome this issue. Among reconstruction techniques (posterior only: PR; anterior only: AR; total: TR), TR facilitates a faster and higher continence recovery compared to standard approach or PR or AR only (40). Moreover, all of the open RP are generally accompanied by a high risk of developing *de novo* incontinence and patients may need further interventions. In such cases, subsequent artificial urinary sphincter implantation is the most common treatment option with the best available evidence (39). In the future, artificial urinary sphincter implantation or TR should be encouraged to decrease the complication of incontinence after RP. Further evaluation of the effect of artificial urinary sphincter implantation or TR for UI after RP would be important in the future to clarify the differences of UI after RP or IMRT. In addition, the RP group had a higher risk of hernia in the long term, which is consistent with previous reports (41–43). Despite evidence of hormone effects on the development of inguinal hernia (44), no study has evaluated the association between hernia risk and IMRT+HT. To the best of our knowledge, this is the first population-based study to show that IMRT+HT therapy has a lower hernia risk than RP. Our findings support that high-dose IMRT combined with long-term HT might be more beneficial than RP for reducing the acute, subchronic, and chronic complications of urine dysfunction and hernia (**Tables 1**, **2**).

BPH symptoms among older people diagnosed with HR-LPC have not been evaluated in different therapeutic modalities. The older age, the higher prevalence of BPH in men with PC (29). Our results indicated that the RP group had lower odds of BPH symptoms than the IMRT+HT group. Our finding is reasonable because RP might involve the removal of most of PC and tissue relative to IMRT. Therefore, BPH symptoms should be higher in the IMRT group, because most prostate tissue was reserved in the IMRT group (**Tables 1**, **2**). Recent studies have reported inconsistent findings on the obstructive urine symptom resulting from treatment in patients with LPC. Hamdy et al. reported that obstructive urinary symptoms were more common in patients undergoing RT than in those undergoing RP (32, 45). By contrast, Lennernas reported no difference between different treatment modalities (46). Ours is the first study to report that RP contributes to lower BPH risk in older men with HR-LPC compared with IMRT+HT. Further RCTs are necessary to identify the effects of treatments on BPH symptoms in older men with HR-LPC.

Our study is the first long-term follow-up study with a large sample size to report acute, subchronic, and chronic posttreatment complications between patients undergoing RP and high-dose IMRT+HT. Our study has strengths. First, we balanced clinic pathological characteristics among the different modalities to ensure consistency in the complexity and difficulties of the various treatment options. PSM enabled us to extract the true association between the acute, subchronic, and chronic complications and therapeutic models. Second, this is the first study to evaluate the acute, subchronic, chronic complications of RP and IMRT in older men with HR-LPC. Finally, our findings indicated that IMRT+HT has more favorable outcomes in terms of UI and hernia. By contrast, the RP group had a significantly lower risk of BPH symptoms at all time points and a lower risk of impotence during the first year after treatment.

Our study has some limitations. This was a retrospective observational study in which treatment options for patients were mainly selected based on clinician or patient preferences. Although we used PSM, other confounders may still exist. Furthermore, because our study cohort comprised an Asian population, our findings should be interpreted cautiously for other ethnicities.

## Conclusions

In this retrospective population-based cohort study including older men with HR-LPC, high-dose IMRT combined with long-term HT was associated with lower odds of UI and hernia than RP. However, RP was associated with better outcomes for BPH symptoms throughout the follow-up period and for impotence during the first year after treatment. Thus, the study provides valuable references for shared decision-making for guiding optimal therapy selection for older men with HR-LPC diagnosed as per NCCN guidelines.

## Data Availability Statement

The raw data supporting the conclusions of this article will be made available by the authors, without undue reservation.

## Ethics Statement

The study protocols were reviewed and approved by the Institutional Review Board of Tzu-Chi Medical Foundation (IRB109-015-B). Written informed consent for participation was not required for this study in accordance with the national legislation and the institutional requirements.

## Author Contributions

Conception and design, S-YW, LH, CL, and C-CH; Collection and assembly of data, S-YW and C-CH; Data analysis and interpretation, S-YW; Administrative support, S-YW; Manuscript writing, S-YW, LH, CL, and C-CH; Final approval of manuscript, all authors. All authors contributed to the article and approved the submitted version.

## Funding

Lo-Hsu Medical Foundation, LotungPoh-Ai Hospital, supports S-YW’s work (Funding Number: 10908, 10909, 11001, 11002, 11003, 11006, and 11013).

## Conflict of Interest

The authors declare that the research was conducted in the absence of any commercial or financial relationships that could be construed as a potential conflict of interest.

## Publisher’s Note

All claims expressed in this article are solely those of the authors and do not necessarily represent those of their affiliated organizations, or those of the publisher, the editors and the reviewers. Any product that may be evaluated in this article, or claim that may be made by its manufacturer, is not guaranteed or endorsed by the publisher.

## Abbreviations

PC, prostate cancer; HR-LPC, high-risk localized prostate cancer; RP, radical prostatectomy; RT, radiotherapy; IMRT, intensity-modulated radiotherapy; HT, hormone therapy; aOR, adjusted odds ratio; OR, odds ratio; CI, confidence interval; AUA, American Urological Association, EAU, European Association of Urology; NCCN, National Comprehensive Cancer Network; PSA, prostate-specific antigen; PSM, propensity score matching; NHIRD, National Health Insurance Research Database; TCRD, Taiwan Cancer Registry Database; AJCC, American Joint Committee on Cancer; ADT, androgen-deprivation therapy; ICD-9-CM, International Classification of Diseases, Ninth Revision, Clinical Modification; CCI, Charlson comorbidity index; RCT, randomized controlled trial; T, tumor; BPH, benign prostatic hyperplasia; UI, urinary incontinence; EBRT, external beam radiotherapy; SD, standard deviation.
