# Supplementary material for: Acute, Subchronic, and Chronic Complications of Radical Prostatectomy Versus Radiotherapy With Hormone Therapy in Older Adults With High-Risk Prostate Adenocarcinoma
Source: Front Oncol. 2022 May 2;12:875036. doi: 10.3389/fonc.2022.875036 (PMC9108927; doi:10.3389/fonc.2022.875036)
Supplement: Supplementary file 1 [file Table_1.docx]

**TABLE S1.** Characteristics of older men aged ≥80 years with high-risk prostate adenocarcinoma stratified by whether they underwent RP or IMRT+HT

|  | | RP N = 277 | | IMRT + long-term HT N = 382 | |  |
| --- | --- | --- | --- | --- | --- | --- |
| Covariates |  | n | (%) | n | (%) | *P* value |
| Age | Mean (SD) | 84.6 | (3.2) | 85.1 | (3.5) | .1098 |
|  | Median (Q1–Q3) | 84 | (82–86) | 85 | (82–87) |  |
|  | 80–89 | 256 | (92.4) | 340 | (89.0) | .9090 |
|  | 90+ | 21 | (7.6) | 42 | (11.0) |  |
| Year of diagnosis | 2011–2012 | 47 | (17.0) | 60 | (15.7) | .9837 |
|  | 2013 | 49 | (17.7) | 72 | (18.8) |  |
|  | 2014 | 57 | (20.6) | 82 | (21.5) |  |
|  | 2015 | 62 | (22.4) | 83 | (21.7) |  |
|  | 2016 | 62 | (22.4) | 85 | (22.3) |  |
| CCI scores | 0 | 102 | (36.8) | 145 | (38.0) | .7626 |
|  | 1 | 77 | (27.8) | 104 | (27.2) |  |
|  | 2+ | 98 | (35.4) | 133 | (34.8) |  |
| Myocardial infarction |  | 4 | (1.4) | 7 | (1.8) | .8348 |
| Congestive heart failure |  | 10 | (3.6) | 13 | (3.4) | .7740 |
| Peripheral vascular disease |  | 10 | (3.6) | 10 | (2.6) | .4913 |
| Cerebrovascular disease |  | 31 | (11.2) | 48 | (12.6) | .8997 |
| Chronic pulmonary disease |  | 41 | (14.8) | 57 | (14.9) | .7090 |
| Diabetes |  | 73 | (26.4) | 94 | (24.6) | .6732 |
| Hypertension |  | 166 | (59.9) | 233 | (61.0) | .9782 |
| Income | <NTD 21,000 | 92 | (33.2) | 119 | (31.2) | .8053 |
|  | NTD 21,000–30,000 | 124 | (44.8) | 170 | (44.5) |  |
|  | NTD 30,000–45,000 | 29 | (10.5) | 41 | (10.7) |  |
|  | NTD 45,000+ | 32 | (11.6) | 52 | (13.6) |  |
| Hospital area | North | 128 | (46.2) | 169 | (44.2) | .9637 |
|  | Central | 82 | (29.6) | 115 | (30.1) |  |
|  | South | 61 | (22.0) | 89 | (23.3) |  |
|  | East | 6 | (2.2) | 9 | (2.4) |  |
| Hospital level | Medical center | 178 | (64.3) | 244 | (63.9) | .5094 |
|  | others | 99 | (35.7) | 138 | (36.1) |  |
| Clinical T stage | T1 | 96 | (34.7) | 128 | (33.5) | .9366 |
|  | T2a | 57 | (20.6) | 77 | (20.2) |  |
|  | T2b | 26 | (9.4) | 44 | (11.5) |  |
|  | T2c | 89 | (32.1) | 120 | (31.4) |  |
|  | T3a | 9 | (3.2) | 13 | (3.4) |  |
| Gleason score | ≤5 | 0 |  | 0 |  | .9570 |
|  | 6 | 19 | (6.9) | 33 | (8.6) |  |
|  | 7 | 187 | (67.5) | 253 | (66.2) |  |
|  | 8 | 44 | (15.9) | 64 | (16.8) |  |
|  | 9+ | 22 | (7.9) | 23 | (6.0) |  |
|  | Missing | 5 | (1.8) | 9 | (2.4) |  |
| Grade (max Gleason grade) | 1-2 | 5 | (1.8) | 9 | (2.4) | .9624 |
|  | 3 | 19 | (6.9) | 32 | (8.4) |  |
|  | 4 | 223 | (80.5) | 304 | (79.6) |  |
|  | 5 | 30 | (10.8) | 37 | (9.7) |  |
| Preoperative PSA, ng/mL | Mean (SD) | 11.7 | (7.6) | 12.7 | (11.6) | .1942 |
|  | Median (IQR) | 10.3 | (6.8–15.1) | 10.5 | (7.2–14.9) |  |
|  | 0–5 | 22 | (7.9) | 34 | (8.9) | .9770 |
|  | 5–10 | 95 | (34.3) | 116 | (30.4) |  |
|  | 10–20 | 109 | (39.4) | 158 | (41.4) |  |
|  | 20+ | 15 | (5.4) | 28 | (7.3) |  |
|  | Missing | 36 | (13.0) | 46 | (12.0) |  |
| D’Amico risk classification | Localized intermediate | 125 | (45.1) | 171 | (44.8) | .8449 |
|  | Localized high | 140 | (50.5) | 194 | (50.8) |  |
|  | Localized advanced | 12 | (4.3) | 17 | (4.5) |  |
| Follow-up time, months | Mean (SD) | 61.7 | (18.4) | 58.4 | (18.9) | .8742 |

RP, radical prostatectomy; IMRT, intensity-modulated radiation therapy; IQR, interquartile range; SD, standard deviation; T, tumor; PSA, prostate-specific antigen; N, number; HT, hormone therapy; NTD, new Taiwan dollars
